# Cholecystokinin Revisited: CCK and the Hunger Trap in Anorexia Nervosa

**DOI:** 10.1371/journal.pone.0054457

**Published:** 2013-01-17

**Authors:** Ulrich Cuntz, Paul Enck, Erich Frühauf, Peter Lehnert, Rudolf L. Riepl, Manfred M. Fichter, Bärbel Otto

**Affiliations:** 1 Klinik Roseneck - Center for Behavioral Medicine, Prien, Germany; 2 Paracelsus Medical University, Salzburg, Austria; 3 Department of Internal Medicine VI: Psychosomatic Medicine and Psychotherapy, University Hospital, Tübingen, Germany; 4 Medical Department - Innenstadt, University Clinic of Munich, Munich, Germany; University of Santiago de Compostela School of Medicine - CIMUS, Spain

## Abstract

**Objective:**

Despite a number of studies in the past decades, the role of Cholecystokinin (CCK) in anorexia nervosa (AN) has remained uncertain. In this study a highly specific assay for the biologically active part of CCK was used in patients with bulimic as well as with the restricting type of AN who were followed over the course of weight gain.

**Methods:**

Ten patients with restricting and 13 with bulimic AN were investigated upon admission (T0), after a weight gain of at least 2 kg on two consecutive weighting dates (T1), and during the last week before discharge (T2) from inpatient treatment in a specialized clinic. Blood samples were drawn under fasting conditions and 20 and 60 minutes following a standard meal (250 kcal). Data were compared to those of eight controls matched for sex and age. Gastrointestinal complaints of patients were measured by a questionnaire at each of the follow-up time points.

**Results:**

At admission, AN patients exhibited CCK-levels similar to controls both prior to and after a test meal. Pre and post-meal CCK levels increased significantly after an initial weight gain but decreased again with further weight improvement. CCK release was somewhat lower in bulimic than in restricting type AN but both subgroups showed a similar profile. There was no significant association of CCK release to either initial weight or BMI, or their changes, but CCK levels at admission predicted gastrointestinal symptom improvement during therapy.

**Conclusions:**

Normal CCK profiles in AN at admission indicates hormonal responses adapted to low food intake while change of eating habits and weight gain results in initially increased CCK release (counteracting the attempts to alter eating behavior) that returns towards normal levels with continuous therapy.

## Introduction

Circulating cholecystokinin (CCK) represents a group of peptides of different length that are a cleavage product of preprocholecystokinin. It is released in the gastrointestinal tract as well as in the central nervous system. Whereas the unsulfated tetrapetide CCK-4 is active at the CCK-B receptors that are predominantly found in the brain, the CCK-forms that bind to the A-type receptor of the gastrointestinal tract are all sulfated and comprise CCK-8-S, CCK-33-S, CCK-39-S and CCK-58-S. Satiety and meal size limitation is mediated mainly by the CCK-A-receptor [1], [2] and therefore by the sulfated forms of CCK. CCK is very similar in structure to gastrin, so that the last five C-terminal amino acids are the same as those of gastrin.

In the past decades, CCK had been a “hot topic” in the field of eating disorder research since it had been shown that CCK induces satiety and limits meal size in rats [3] and monkeys [4] and it became evident that CCK is relevant for satiation in human subjects as well [5]–[7]. Nevertheless, the role of CCK in the pathogenesis of eating disorders is still far from being known. Two studies [8], [9] found higher CCK levels in anorexia nervosa (AN) patients compared to controls, whereas three others [10]–[12] did not. Patients with bulimia nervosa and with bulimic-type AN had lower CCK release than patients with restricting type AN [10], [11]. These conflicting data may at least in part be due to the fact that all these studies were not able to determine sulfated, bioactive CCK: The assays used in previous studies showed a considerable cross-reaction with gastrin, and given the tenfold higher plasma concentration of gastrin the so far available evidence for a role of CCK in AN is open to debate.

We have established a sensitive radioimmunoassay that is highly specific for the sulfated CCK-subunits active at the CCK-A receptor and shows no cross-reaction with unsulfated and sulfated gastrin [13].

Our study had the following objectives:

1. The first objective of the study was to determine whether patients with AN compared to normal-weight controls have different basal or stimulated CCK-levels. According to some of the previous studies we expected patients with AN to exhibit higher CCK levels and higher meal-induced CCK release than healthy volunteers at admission.
2. According to common clinical experience, it appears to be specifically difficult to increase food intake of AN patients during the initial period of weight gain, while any further weight gain seems comparably easy. We suspected therefore a role of CCK in inducing a premature feeling of satiety while eating during the early phase of therapy. This would show in a higher and increased rise from baseline to post-meal of CCK after an initial weigh gain, as compared to the CCK response of healthy subjects and of the same patients at the time of discharge.
3. We expected restricting AN patients to show higher CCK release than bulimic AN patients. Lower CCK levels in bulimic as compared to restricting AN patients would explain that bulimic AN patients feel less satiation by food intake and thus are vulnerable to loose control during binges.
4. A synergistic effect of CCK and Leptin in the decrease of food intake has been demonstrated suggesting a contribution of CCK to weight control [14], [15]. Rats lacking the CCK-A receptor gene showed disordered eating and increased body weight [16], while knock-out mice lacking the same receptor grow to normal weight [16], [17]. A further aim of the study was therefore to determine the role of endogenous CCK in weight regulation. If CCK is important for the weight regulation in AN, we should expect a significant association of changes in CCK release to changes in BMI.
5. CCK plays a role in the pathogenesis of gastrointestinal disorders [18]–[20]. Gastrointestinal complaints are extremely common in patients with eating disorders, especially in anorexia nervosa [21], [22], and many patients with functional bowel disorders exhibit signs of eating disorders [23]. We therefore investigated whether CCK-release is related to the degree of gastrointestinal complaints in AN patients.

## Materials and Methods

### Participants

Patients with a diagnosis of anorexia nervosa according to the *Diagnostic and Statistical Manual of Mental Disorders (DSM IV)* [24] (main criteria: body weight less than 85% of that expected, intense fear of gaining weight, body image disturbance, amenorrhea in females) admitted to the Klinik Roseneck, Prien, Germany, a center for behavioral medicine for treatment of AN were asked to participate in the study. All patients took part to a multi-modal cognitive behavioral inpatient treatment program. Eating behavior was regulated by contract management and guidance by therapists. After complete description of the study to the subjects, written informed consent was obtained at least one day before the start of the study. Ethical approval was provided by Ludwig Maximilian University of Munich ethics committee.

Tests were performed in patients at the following time points:

T0: within the first days after admission (no reported weight gain in the last two weeks prior to and no measured weight gain since admission);

T1: after an initial weight gain of at least two kg for two consecutive weighing events with the patients weighted twice weekly;

T2: during the last week before discharge after a minimal weight gain of 4 kg.

Eight healthy and sex and age-matched volunteers were measured twice three weeks apart, and five of them at another occasion for a third time.

All participants were studied in the morning between 07.00 and 08.00 h after fasting overnight (12 h) to account for circadian variability of CCK-release [25]. Ten ml blood samples were drawn from a forearm vein using an indwelling catheter. We also determined body weight, Body Mass Index (BMI = weight/height^2^), and body composition by multi frequency bio-impedance method (InBody 520, Bio-Space - JP Global Markets, Eschborn, Germany).

Subsequently, all received a 250 ml standard liquid meal containing 250 kcal (9.4 g protein, 34.4 g carbohydrates, and 8.3 g fat) (Salvimulsin®, Nestle Nutrition GmbH, Frankfurt, Germany) to be consumed within 5 minutes. Postprandial blood samples were taken 20 and 60 minutes after the intake of the meal.

Complete data were available from 23 anorectic patients (22 females; 10 restricting, 13 binge-purging type according to DSM IV criteria) who completed all three measurements and gained enough body weight to meet inclusion criteria.

All patients completed a questionnaire for gastrointestinal complaints at T0, T1 and T2. This instrument assesses gastrointestinal complaints in functional gastrointestinal syndromes [26]. The questionnaire measures the frequency of 30 gastrointestinal symptoms on a four-point rating scale from ‘never’ to ‘nearly always’ and subjective impairment by these symptoms on a five point rating scale between ‘not at all’ to ‘very badly’. A total GI score (GIS) was computed as the sum of all 30, ranging between 0 and 90, and a gastrointestinal impairment score (GIM) ranging between 0 and 100.

### CCK Measurement

Plasma CCK concentrations were determined by a double-antibody radioimmunoassay. The antiserum (CH40IX) was produced by immunization of rabbits with sulfated CCK-8 (Sigma Chemie, Munich, Germany) coupled to bovine serum albumin (Paesel Frankfurt/Main, Germany). The antibody (K_eff_ 6.6×10^11^ l/mol) is specifically directed to the biologically active site of CCK including the sulfated tyrosil(yl) residue at position 7 from the C-terminal end. It shows no cross reactivity with unsulfated CCK-8 or other unsulfated molecular forms of CCK and unsulfated gastrin-17 or –34 and a cross reactivity of less than 1% with sulfated gastrin. As the antiserum showed an equipotent binding to CCK-8-S and also to other sulfated (biologically active) molecular forms of CCK, the term ‘CCK-like immunoreactivity (CCK-LI)` has been introduced [27].

### Statistics

Statistics were calculated using SPSS Version 19.0 software. Analyses of meal effects and their changes over time were performed by 3×3 repeated measures ANOVA with the within-factors “time” (admission, plus 2 kg, discharge) and “meal” (baseline, post 20 min, post 60 min). Data from healthy volunteers were used to outline the normal range of CCK release prior to and following the meal. To test the effects of AN type, we added the between-factor “diagnosis” (restricting AN, bulimic AN) in a second analysis step. Post-hoc t-tests or chi^2^ tests were used to compare AN values to values of normal volunteers and between AN subgroups for different time point. Associations between CCK levels and clinical measures were assessed by Pearsońs correlation coefficient. Effect size was computed as eta^2^ and provides an estimate of the explained variance (in percent). All data are given in mean ± SD or SEM, and the significance level was set to 0.05 for all tests.

## Results

Baseline characteristics of patient and volunteers are given in Table 1. As can be seen controls and AN patients are comparable with respect to gender and age. Improvement of eating behavior is illustrated by a significant weight gain in patients.

**Table 1:** Anthropometric and clinical data at T0 (admission for anorexia nervosa, first measurement for controls) (n.s. =  not significant).

	Anorexia	Controls	Statistics
N	23	8	
Females	22	7	n.s.
Age (mean)	25.3±7.6	30.5±6.6	n.s.
Weight (kg)	40.9±4.8	62.2±6.3	p<.001
Height (cm)	164.5±6.4	170.7±5.5	p = .019
BMI (kg/m^2)^	15.1±1.4	21.3±1.6	p<.001
Fat (%)	16.4±17.4	25.9±7.4	n.s.
Fat mass (kg)	5.4±2.1	16.1±5.2	p = .01
Lean body (kg)	36.6±3.3	46.1±6.9	p<.001
Baseline CCK (µmol/ml)	1.9±2.4	2.1±1.7	n.s.
GIS*	35.7±12.5	6.8±2.8	p<.001
GIM^+^	34.8±14.7	4.4±3.2	p<.001
Weight gain (kg) (T2– T0)	7.7±3.1	0.7±0.6	p<.001
BMI gain (T2– T0)	2.5±0.9	0.001±0.3	p<.001

* Gastrointestinal symptom score; +: Gastrointestinal impairment score.

Patients as well as controls received a nutrition base on the recommendations of the German Nutrition Association (DGE) containing 55–60% carbohydrates. Caloric intake was adjusted in anorectic subjects whenever possible with the aim to gain approximately 100 g per day.

Mean duration between admission (T0) and 2 kg of weight gain (T1) was 26.4 (SD: 12.2, range: 8 to 48) days. Mean duration from T1 to discharge (T2) was 50.2 (SD: 17.8, range: 22 to 83) days.

### Meal-induced CCK-LI Release in Healthy Participants

**Figure 1:**
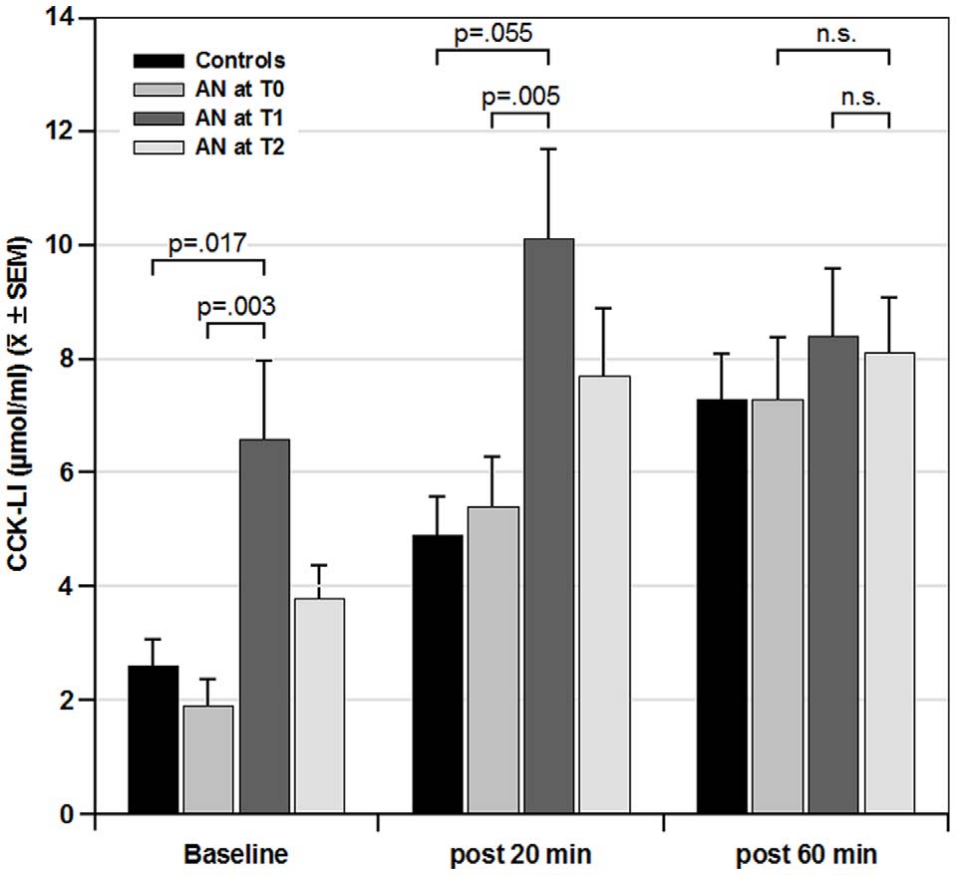
CCK-LI values (µmol/ml) (mean ± SEM) in control volunteers and patients with anorexia nervosa (AN) at time points (T0, T1, T2) during inpatient treatment. Data are groups for baseline (pre-meal) (left), and 20 min (middle) and 60 min (right) following a standard test meal of 250 kcal. A 3×3 ANOVA for data from AN patients revealed significant main effects of “meal” and “time” and a “meal×time” interaction. Post-hoc unpaired t-tests for comparison of data from healthy volunteers to AN patients at T1 were significant for baseline and 20 min post meal but not for 60 min post-meal. Post-hoc paired t-tests for data from AN patients between T0 and T1 were significant for baseline and 20 min post meal but not for 60 min post-meal.

Controls (n = 8) who did not change weight (Table 1) showed a constant CCK release for T0 and T1 (main effect of time F = 0.303, p = .599, n.s., eta^2^ = .042) but a significant increase from baseline with the test meal (main effect of meal: F = 20.63, p<.001, eta^2^ = .74). The means and standard error (SEM) at T0, T1, and T2 defined the normal range for comparison to AN (see Figure 1, below).

### Anorexia at Admission Versus Controls (Hypothesis 1)

Immediately after admission (T0), CCK-LI levels were within the normal range of the controls prior to the meal. The test meal led to a highly significant rise in plasma CCK-LI concentration 20 and 60 min thereafter (main effect of meal: F = 18.46, p<.001, eta^2^ = .45) and did not exceed the normal response (Figure 1).

### Anorexia during the Clinical Course (Hypothesis 2)

Compared to measures at admission (T0), CCK-LI release was significantly increased after an initial weight gain of 2 kg (T1) but this effect was nearly reversed at the time of discharge (T2) (main effect of time: F = 7.271, p = .002, eta^2^ = .24) (see Figure 1).

Post-hoc t-test comparison of the pre-meal CCK-LI levels between normal controls and AN patients at T1 revealed significantly (p = .017) increased values for AN, while differences in post-20-min values at T1 did not quite reach significance levels. Post-hoc paired t-tests in AN patients for CCK-LI release between T0 and T1 were significant for baseline (p = .003) and 20 min post (p = .005) but not for 60 min post-meal.

A significant “meal×time” interaction (F = 2.519, p = .047, eta^2^ = .10) indicates a higher meal-induced CCK-LI-release at T1 and T2 as compared to T0 in AN patients. At T2, meal-induced CCK-LI-release was still higher in AN as compared to the control values 20 min after the meal, while the 60-min level had returned to control values.

Adding the duration (T0 to T1, in days) for the initial 2 kg weight gain as a covariate to the ANOVAs did not change any of the found significances, nor did the duration itself have an effect of CCK-LI release (data not shown).

### Restricting versus Bulimic AN Patients (Hypothesis 3)

**Figure 2:**
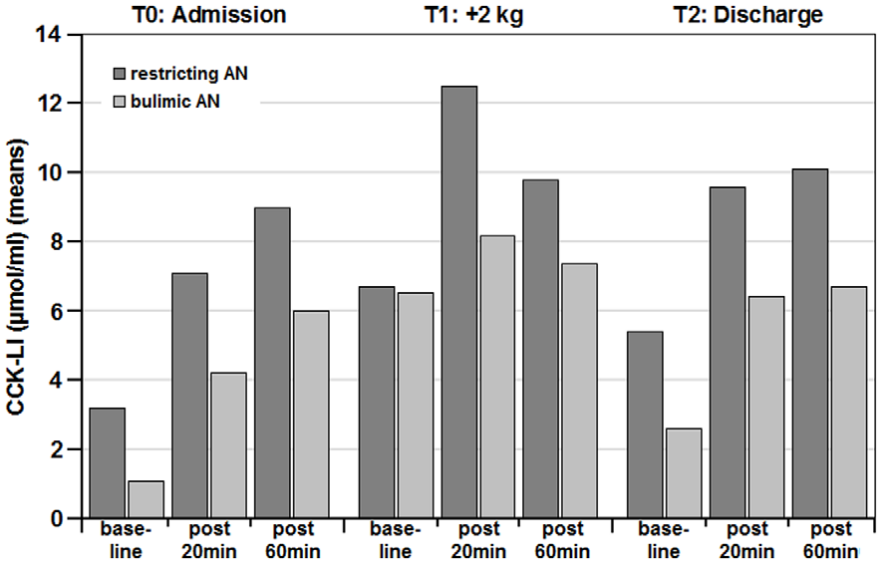
CCK-LI values (µmol/ml) (means) in patients with restricting (n = 10) or bulimic types (n = 13) of anorexia nervosa (AN) pre-meal and 20 min and 60 min post-meal. Data are grouped for time points during inpatient treatment: left: T0 = at admission; middle: T1 = after weight gain of ≥2 kg; right: T2 = at discharge). ANOVA for the between-group effect “diagnosis” did not reach significance.

While CCK-LI release was lower for all measurements in bulimic AN as compared to restricting AN (Figure 2) this effects did not reach significance levels (main effect of diagnosis: F = 2.975, p = .099, eta^2^ = .12).

The factor “diagnosis” (restricting, bulimic) did not interact with the meal-induced CCK-LI release at any of the measurement points (T0 to T2). Excluding the only male AN patient did not change the results.

### CCK-LI Release and Weight, BMI, and Weight and BMI Gain (Hypothesis 4)

Baseline CCK-LI release was neither correlated to BMI nor to body weight at T0. It was also not significantly associated with the change in weight and BMI during therapy (data not shown).

### CCK-LI Release and Gastrointestinal Symptoms (Hypothesis 5)

Symptom severity (GIS) constantly improved between T0 and T2 in both patient groups (main effect of time: F = 23.03, p<.001, eta^2^ = .638), with no group difference (main effect of diagnosis: F = 0.745, n.s.). Similarly, the GIM score improved irrespective of the diagnostic subgroups (F = 9.171, p<.001, eta^2^ = .304).

Among the GI symptoms, only the frequency of vomiting was significantly but moderately correlated to basal and stimulated CCK-LI plasma concentrations at admission across all AN patients (pre-meal: r = .33, p<.05; 20 minutes post meal: r = .41, p<.01; 60 minutes post meal: r = .31, p<.05).

**Figure 3:**
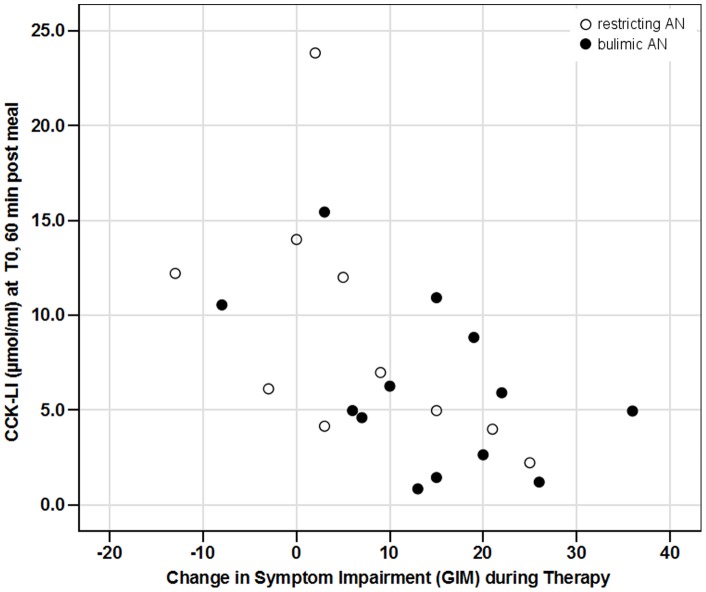
Bivariate correlation between CCK-LI values (µmol/ml) at T0 (admission) 60 min after test meal intake in AN patients (open circles: restricting AN; closed circles: bulimic AN), and the change in gastrointestinal symptom impairment (GIM) score between T0 and T2 (discharge). Pearsońs correlation coefficient (r = −.537, p = .008) indicates that patients with high CCK-LI after the meal have significant lower improvement of GIM during the subsequent inpatient treatment. The same association was found for baseline and 20 min post-meal CCK-LI levels, and for gastrointestinal symptom severity (GIS) improvement.

Baseline CCK-LI release was also not significantly correlated with the GIS but correlated negatively with GIM score at T0 (r = −.48, p = .02). However, CCK-LI values at T0 predicted changes in GIS and GIM scores from T0 to T2: the higher the CCK-LI values at baseline and 20 and 60 min post meal at T0, the lower were the GIS and GIM improvements during therapy (all correlation coefficients between r = −.417 and r = −.537, p = .047 to p = .008). Figure 3 shows the strongest association between CCK-LI at T0, 60 min post-meal and the GIM change between T0 and T2 (r = −.537, p = .008).

At the time of discharge (T2), the associations between CCK-LI and GIS/GIM scores were no longer significant (data not shown).

## Discussion

With our study, we found the following – in part surprising – results:

1. In patients with AN, baseline as well as meal-induced CCK-LI release is well within the range of normal values when compared to values obtained from healthy normal-weight volunteers (see Figure 1). This is against our expectation (Hypothesis 1) and in part against current published data.

In animals (rats) elevated CCK plasma concentrations and down-regulated CCK-A receptors after prolonged food restriction of more than 20 days were found [28]. After short-term fasting for only three days, CCK levels were decreased in rats in another study [29]. In humans, two studies [8], [9] found higher CCK levels in anorexia nervosa (AN) patients compared to controls, whereas three others [10]–[12] did not. More recent studies found a decrease of CCK in AN [30] but an increase in undernourished women [31]. In obese humans no changes in CCK release were found after 10 weeks of dieting [32]. Binge-eating, however may cause an adaptive lowering of CCK release to large amounts of food, which can be observed in the same way in normal weight binge eaters [33]. Discrepancies especially in humans may in part be explained by the fact that all these studies did not assess sulfated bioactive CCK but rather assays with a considerable cross-reaction with gastrin, and in light of the ten-fold higher plasma concentration of gastrin the evidence for a role of CCK in AN remains unclear.

Our data indicate that CCK-LI release in long-term and severe anorexia nervosa (irrespective of the subtype) is well adapted to the chronic malnutrition and responds normally when stimulated with a standard (test) meal of low volume and caloric content (250 kcal).

1. A moderate (2 kg) increase in weight achieved during controlled inpatient treatment for a few days to weeks results in a significant increase of both baseline as well as meal-induced CCK-LI release that exceeds the response of healthy volunteers on average by nearly 100%. This has not been observed previously and may in part be explained the novel measure of biologically active CCK that was applied here for the first time.

According to common clinical experience, such a strong response to moderate food intake may be able to limit and counteract the therapeutic attempts to further gain weight. It may explain the increasing difficulties that anorectic patients perceive in the first weeks of weight gain to maintain a sufficient food intake. This phenomenon may be called a “hunger trap”: Once the CCK system has adapted to low food intake, it self-limits weight gain by increasing satiety even with moderate food intake. Only with further weight gain the CCK release returns to the initial and normal state (Hypothesis 2). High postprandial CCK levels were observed in girls with AN [34] and were made responsible for the aggravation of the course of the disease via intensifying nausea and vomiting.

1. We found a consistent but small difference in CCK-LI release between restricting and bulimic AN patients, with bulimic patients exhibiting overall lower values (see Figure 2). This supports our Hypothesis 3, and the missed significance may in part be due to small sample sizes in the restricting (n = 10) and bulimic (n = 13) subgroup.

Lower CCK-LI levels in bulimic as compared to restricting AN patients would explain that bulimic AN patients feel less satiety after eating and thus are vulnerable to loose control during binges. Binge-eating may cause an adaptation of CCK release to large amounts of food, which can be observed in the same way in normal weight binge eaters [33]. However, CCK-LI release with a meal was closer to the normal ranges than in restricting AN patients in our study and thus questions this explanation.

Differences between restricting and bulimic food intake on CCK release have not previously been described in other experimental studies, but it needs to be kept in mind that there is however no adequate animal model for bulimic eating behavior.

As to expect, bulimic patients had a higher frequency of vomiting at admission but the difference vanished during the course of therapy and therefore cannot explain the difference between restricting and bulimic patients throughout the therapy. It has been reported [35] that percent body fat is higher in bulimic AN than in restricting AN which we confirm here (see Table 2). Bulimic AN patients also had higher body weight at admission. Whether this limits the CCK-LI release – despite similar body weight and BMI (see Table 2) – in comparison to restricting AN patients has to remain open but warrants further studies.

**Table 2:** Anthropometric and clinical data between restricting and bulimic anorexia nervosa patients and changes between admission and discharge (n.s. = not significant).

	Restricting Anorexia	Bulimic Anorexia	Statistics
N	10	13	
Females	10	12	n.s.
Age (mean)	26.1±7.2	24.7±8.1	n.s.
Weight (kg)	38.7±3.9	42.6±4.9	p<.05
Height (cm)	162.9±5.2	165.7±7.1	n.s.
BMI	14.6±1.4	15.5±1.4	n.s.
Fat (%)	11.2±3.6	20.4±22.4	n.s.
Fat mass (kg)	4.4±1.8	6.2±1.9	p = .031
Lean body (kg)	34.2±2.6	36.6±3.5	n.s.
GIS*	31.4±12.8	38.9±11.7	n.s.
GIM+	30.1±16.5	38.5±12.6	n.s.
Weight gain (kg)	6.7±1.9	8.5±3.6	n.s.
BMI gain	2.1±0.7	2.9±1.0	n.s.
GIS improvement	8.6±7.9	15.8±10.6	n.s.
GIM improvement	6.4±11.4	14.2±11.1	n.s.
Delta CCK-LI (µmol/ml) (baseline)#	3.6±3.7	5.4±8.4	n.s.
Delta CCK-LI (20 min post meal)#	5.4±4.5	4.1±8.8	n.s.

* Gastrointestinal symptom score;

+ Gastrointestinal impairment score;

# CCK increase between T0 and T1.

1. We expected the CCK-LI release before and after a meal to be associated with weight, BMI, and weight and BMI gain during therapy (Hypothesis 4). While human data are not available on this issue, animal data are conflicting: Rats lacking the CCK-A receptor gene showed disordered eating and increase in body weight [16], while knock-out mice lacking the same receptor grow to normal weight [16], [17].

We found a supporting but moderate association (explaining 25% of variance) (see Figure 3) between the starting weight and the increase in pre-meal CCK-LI between the initial measurement (T0) and after a moderate weight gain (T1) but no further associations of the initial CCK-LI levels to weight and BMI changes. It is thus not likely that CCK is involved in the normal process of weight regulation but further studies are needed.

5. CCK release is associated with symptoms in normal weight gastrointestinal disorders and induces motility disturbances associated with complaints [20], [23], [36]. On the one hand, gastrointestinal symptoms are frequent in AN (see Table 1) [21], [22]; on the other hand, patients with functional bowel disorders frequently exhibit signs of eating disorders [23]. We therefore expected associations between gastrointestinal symptoms and CCK-LI release, specifically at the initial measurement point (T0) (Hypothesis 5).

Both the gastrointestinal symptom score (GIS) as well as the subjective impairment by these symptoms (GIM) were moderately associated with CCK levels at T0, and with both pre as well as post-meal levels (Figure 3). With increasing body weight, normalizing CCK-LI response, and decreasing GIS and GIM scores, these associations were no longer significant at T2. This may indicate that the association between CCK response and gastrointestinal symptoms may be strong in disordered eating and/or digestion, but may not be related to each other when both return to normal functioning. It may also indicate that AN patients who do not adapt their CCK level to low food intake (see above) represent a – potentially genetically determined [37] - subgroup of AN patients that are at risk to not respond to weight gain [38]. This may also link CCK to further and specifically neuropsychological functions in AN [39]–[42]. Further research is needed to substantiate this hypothesis.

### Summary

Anorectic patients, specifically of restricting-type AN show well-adapted CCK-release pattern with stable but abnormal eating behavior and under-nutrition. With the initiation of weight gain, an initial exaggerated CCK-response occurs that - clinically - may terminate further weight gain by inducing premature satiety (“hunger trap”). Only if therapy can overcome this intrinsic handicap, further weight gain will lead to normalization of CCK levels again. Patients who do not adapt to the normalization of CCK are at risk to experience high levels of gastrointestinal symptoms that do not improve with weight therapy.
